# Linking autoimmunity to the origin of the adaptive immune system

**DOI:** 10.1093/emph/eoy001

**Published:** 2018-01-12

**Authors:** Robert Bayersdorf, Arrigo Fruscalzo, Francesco Catania

**Affiliations:** 1 Institute for Genome Stability in Aging and Disease, Medical Faculty, University of Cologne, 50931 Cologne, Germany; 2 Institute for Evolution and Biodiversity, University of Münster, 48149 Münster, Germany; 3 Clinic of Obstetrics and Gynecology, St Franziskus Hospital, 59227 Ahlen, Germany; 4 Department of Obstetrics and Gynecology, University Hospital of Münster, 48149 Münster, Germany

**Keywords:** adaptive immune system, innate immune system, natural autoantibodies, self-recognition, pregnancy, immune tolerance

## Abstract

In jawed vertebrates, the adaptive immune system (AIS) cooperates with the innate immune system (IIS) to protect hosts from infections. Although targeting non-self-components, the AIS also generates self-reactive antibodies which, when inadequately counter-selected, can give rise to autoimmune diseases (ADs). ADs are on the rise in western countries. Why haven’t ADs been eliminated during the evolution of a ∼500 million-year old system? And why have they become more frequent in recent decades? Self-recognition is an attribute of the phylogenetically more ancient IIS and empirical data compellingly show that some self-reactive antibodies, which are classifiable as elements of the IIS rather then the AIS, may protect from (rather than cause) ADs. Here, we propose that the IIS’s self-recognition system originally fathered the AIS and, as a consequence of this relationship, its activity is dampened in hygienic environments. Rather than a mere breakdown or failure of the mechanisms of self-tolerance, ADs might thus arise from architectural constraints.

## INTRODUCTION

Jawed vertebrates are protected from invading pathogens or pathogenic compounds by two multilayered and synergistic mechanisms: the Innate Immune System (IIS) and the Adaptive Immune System (AIS) [1]. IIS and AIS provide incredibly high levels of protection against external threats. However, these systems may also bear disadvantages that can be life threatening. Healthy autologous or self-components such as DNA, cells or secreted proteins may become targets of antibodies (Abs) that are generated during an immune response thus giving rise to autoimmune diseases (ADs). Self-reactive Abs have long been considered as an unfortunate byproduct of the process of somatic recombination which must be coped with in return for a sophisticated defense system [2]. It has been proposed that mechanisms such as clonal deletion, receptor editing, and anergy (i.e. functional unresponsiveness) [3–6] have evolved to mitigate the negative impacts of self-reactive Abs. On the other hand, self-reactivity can also be non-pathological and crucial for the proper functioning of the AIS [7–10].

Herein we leverage and build on this knowledge to put forward a hypothesis for the emergence of AIS, which proposes that self-recognition was (i) a major driving force in the evolution of a system that would eventually give rise to the current AIS, and (ii) a significant player in imposing constraints on the present-day AIS. It is maintained that the AIS emerged subsequent to the weakening of existing immune defenses, a suboptimal condition that imposed selection pressures favoring further immune defense mechanisms. The proposed hypothesis is uniquely capable of concurrently integrating empirical findings such as the homeostatic function of B-1-derived natural self-reactive Abs and the immunological changes that occur during normal pregnancy with prominent theoretical arguments such as the ‘immunological homunculus’ [8], the ‘immune network theory’ [11–13], the ‘hygiene hypothesis’ [14], and the ‘2R hypothesis’ [15]. Our hypothetical scenario delineates specific changes in the population-genetic environment that occurred around the time when vertebrate lineages diverged from a common ancestor. It also makes testable predictions regarding the mechanistic relationships between the expression/activity of B-1 and B-2-derived Abs.

## INNATE AND ADAPTIVE IMMUNITY

The IIS is a collection of defense measures that provide immediate protection against infections. Widespread across eukaryotes, the IIS acts against conserved pathogen-associated patterns (e.g. lipopolysaccharides of bacterial cell walls). It involves a diverse range of assets (e.g. macrophages, neutrophils, complement system etc.) and employs genetically encoded receptors (e.g. Toll-like receptors) and secreted proteins [1].

The AIS with its classical immunoglobulins (Igs) and molecules such as the major histocompatibility complex (MHC), on the other hand, is currently restricted to the group of jawed vertebrates. Dependent on IIS elements, the AIS is highly specific and generates a diverse repertoire of receptors during the process of germ-line-to-soma differentiation. When challenged by new pathogens or pathogenic elements, matching receptors are selectively amplified to target the threat and to mount an immune response wherein components of the IIS are also involved. During this process, some of the selected cells are stored as memory cells enabling an accelerated immune response should the pathogen be re-encountered [1].

The diversity of the acquired antibody repertoire is generated by V(D)J recombination [16, 17]. During this developmental process, immune cells known as B and T lymphocytes recombine one copy each of multiple V, (D) and J gene sections in the genomic receptor locus to form functional somatic genes, i.e. B-cell receptors (BCRs) and T-cell receptors (TCRs). Two proteins (*RAG1* and *RAG2*) are required to activate V(D)J recombination [18, 19]. As multiple copies of V, (D) and J segments are present in the germ-line genome, >10^6^ antibody variants can be yielded theoretically for human BCRs by combinatory possibilities, although some diversity restriction may be imposed by the non-random use of individual amino acids in the third complementarity region of the H chain or the biased composition of the antibody repertoire in fetus and adult [20]. This number may rise further as a consequence of the imprecise joining of the V, (D) and J segments [21, 22]. The considerable diversity of receptor variants that result from this process of diversification should provide adequate protection against any new pathogen encountered. However, there is a risk that it may target self-components and harm the host. Auto-reactive cells/receptors may be repressed or selected against at various points during the lymphocytes’ lifetime [23]. Elimination of self-reactive immune cells may not always be complete however. Additionally, self-reactive immune cells can normally be detected in physiological conditions (see section ‘The healthy aspect of self-recognition’ below).

## ADAPTIVE IMMUNITY AND ‘THE CHICKEN AND THE EGG’ CONUNDRUM

Although the deletion of self-reactive BCRs and/or TCRs may make sense from a system viewpoint, the modern AIS’s organization raises questions about the sequence of evolutionary events that shaped it. The AIS requires finely tuned regulation to prevent autoimmunity and to secure functional interactions with the IIS. Although it is possible that the early AIS was unregulated and yet selected for its inherent fitness benefits (e.g. pathogen resistance outweighed the costs of autoimmunity), it seems unlikely that such a sophisticated system could have developed without regulatory mechanisms in place. But how can regulatory elements evolve in the absence of the system? A possible answer to this conundrum is that regulatory mechanisms were already present for a system that originally served (and may continue to serve) different functions than those that the AIS presently plays. In this hypothetical system, primordial lymphocyte cells produced germ-line encoded antibody-like proteins that were at least partly reactive towards self-components. Therefore, control mechanisms could evolve to prevent the emergence of pathogenic self-reactivity. The ability to efficiently recognize foreign antigens would have evolved later in the presence—if not owing to the existence—of this self-reactive cell-producing system. While both T and B cells may have played an equally central role in an evolutionary system where self-recognition is a key step towards the modern AIS, in this article we only focus on B cells and Abs/BCRs as possible key players in AIS evolution. This choice is based mainly on the striking properties of B cells, which are discussed further below.

## THE HEALTHY ASPECT OF SELF-RECOGNITION

A traditional paradigm in immunology is that self-reactive Abs are negatively selected owing to the harm that they may cause the organism. This paradigm has been repeatedly challenged however. For example, it has been found that self-reactivity is a common attribute of T cells [24, 25]. Furthermore, self-reactive BCRs may be even necessary for B cells to develop properly and to form the diverse repertoire of BCRs [26, 27]. Finally, the discovery of naturally occurring autoantibodies or NAAbs challenges the notion that self-recognition is merely deleterious [28]. NAAbs closely resemble the primordial lymphocyte receptors that we envisage in our hypothesis. They are naturally found in the sera of healthy, non-immunized, humans and other vertebrates [29–34], and are also detected in animals raised in germ-free conditions [35, 36]. As opposed to somatically hypermutated Abs that result from active immunization, NAAbs are encoded by rearranged germ-line V(D)J gene segments that are unaltered or only minimally altered [37, 38]. They are typically polyreactive, have low binding affinity, and may recognize a broad spectrum of antigens including non-self and self-antigens (e.g. single-stranded DNA, carbohydrate epitopes) [39, 40]. NAAbs primarily belong to the IgM isotype—the most ancient of the antibody classes, also found in sharks in trans-membrane and secretory forms [41, 42]. It is worth noting that IgM NAAbs from sharks, humans, and other vertebrates show considerable levels of conservation in the overall 3D structure and in some framework regions such as the variable domains VH3-30 and VH3-23 [43]. Abs that retain such conserved regions are often expressed early in fetal development, when the ability to respond to specific antigens is low, and are a part of the repertoire directed against T-independent antigens [20, 44].

NAAbs are produced primarily by self-replenishing B-1a cells (NAAb_B-1a_), a subgroup of B-cell lymphocytes that could be classified as part of the IIS [45, 46]. Although responding to probably innate immune signals [47], B-1a cells do not appear to develop into memory B cells [48, 49], which are crucial for the adaptive immune response. Moreover, after being produced chiefly during the fetal and neonatal period, they persist in the individual and, with increasing age, may be complemented by bone marrow-derived B-1 cells [50, 51]. On the other hand, the majority of B-cell lymphocytes are made of follicular B-2 cells that develop later in life and produce non-self-reactive Abs with high-binding affinities [37, 52]. Interestingly, ‘early’ B-2 cells produce Abs that resemble NAAb_B-1a_ in being positively selected for their ability to bind to self-antigens [26]. On the other hand, when the regulatory mechanisms underperform, developing B-2 cells which undergo negative selection for self-reactivity may also produce class switched and affinity matured self-reactive Abs with pathogenic properties. Hereinafter we will refer to B-2 cell-related self-reactive Abs as *SR-*Ab_B-2_, where *SR* stands for self-reactive. Unlike B-2 cells, which undergo negative selection at a later stage of development [23] to guarantee the proper production of non-self reactive Abs, autoreactive B-1a cells persist [53–55]. Thus, not only may self-reactive cells not be purged, they may also be deliberately produced and, in the case of the B-1a cells, preserved.

It is unlikely, as argued in the immunological homunculus theory [8, 56], that the presence of NAAb_B-1a_ indicates a failure of the mechanisms of self-tolerance or that they are a threat to the organisms. Rather, the production and conservation of NAAb_B-1a_ is presumably linked to their unique functions: they are major contributors to tissue homeostasis. Among the functions that have been ascribed to NAAb_B-1a_ are: (i) acting as first line of defense against bacteria, viruses and other pathogenic agents, (ii) participation in the clearance of apoptotic cells as well as tumor and senescent cells, (iii) modulation of the inflammatory response and (iv) reduction of the risk of tissue damage during an immune response [32, 57–68]. B-1a cell-derived NAAbs may also be involved in the maintenance and regulation of the commensal microbiota [69] and contribute to the enhancement of antigene-specific responses e.g. they can stimulate and regulate T-cell responses [51] as well as modulate specific T-cell functions like cytokine secretion and chemotaxis in certain allograft contexts [70].

NAAb_B-1a_ can also protect from AD development [71–73]. For example, the abundance of IgM-NAAb_B-1a_ generally correlates negatively with the severity of systemic lupus erythematosus, whereas the deficiency of IgM-NAAb_B-1a_ can help accelerate this disease [69, 74]. In addition, IgM-NAAb_B-1a_ treatment reduces atherosclerosis [75, 76]. In contrast, IgM-NAAb_B-1a_ deficiency may lead to atherosclerosis [77]. Last, mice that are deficient in serum IgM-NAAb_B-1a_ display an increased and pathological response to self-components [77, 78]. The protective properties of IgM autoAbs against autoimmunity may lie principally in their ability to inhibit inflammatory responses by recognizing and removing apoptotic cells [60]. NAAb_B-1a_ may also protect from autoimmunity by functionally masking antigenic epitopes [79]. By reducing the amount of unengaged antigen, this operation would lessen the need for the AIS to mount a response.

## FROM NAAB_B-1__A_ TO THE ORIGIN OF THE AIS

The findings that are outlined above suggest that in addition to acting as the first line of defense against pathogens, NAAb_B-1a_ may also be instrumental for the deployment of Abs against foreign antigens [27, 80, 81]. These observations align with the accepted view that IIS and AIS act synergistically to mediate host responses to infection and tissue injury. They also hint that a system that has spawned self-reactive Abs in the ancestor of jawed vertebrates may have predated the emergence of a system of non-self targeting Abs, in a similar vein to previously proposed hypotheses [37, 82–87]. Below, we elaborate further on this idea and put forward a three-step evolutionary scenario for the origin of the AIS. In this scenario, the proliferation of distinct Ig-domain containing receptors facilitated the birth (or the amplification) of a regulatory system that controlled the interactions between these Ab-like receptors and their ligand(s) in early vertebrates. Measures for guaranteeing or optimizing the clearance of antigen-bound Abs evolved subsequently and linked the existing innate immunity circuit to the emergent system of Ab-like proteins. Lastly, the AIS emerged from the system producing non-self-reactive Abs. Although targeting non-self-antigens, the AIS could (and continues to) target occasionally self-components, as testimony to its evolutionary origins. Population-genetic environments where the efficiency of natural selection is reduced may have facilitated these evolutionary innovations by favoring the accumulation of mildly deleterious mutations and the preservation of gene duplicates [88].

## THE BIRTH OF AN IMMUNE REGULATORY SYSTEM

Genes homologous to recombinases that are essential for adaptive immunity (*RAG1* and *RAG2*) were present well before the emergence of the AIS-bearing jawed vertebrates [89]. It is likely then that *RAG1* and *RAG2* were domesticated for BCR and/or TCR assembly in primordial lymphocytes sometime after the split between Hyperoartia (e.g. lamprey) and Gnathostomata (jawed vertebrates).

With the RAG genes in place, Ig genes that contained a set of primordial V, (D) and J-like modules may have yielded distinct Ab-like proteins. Some of these Ab-like proteins would have binding affinity toward self-components (e.g. cytokines), as many modern Ig domain-containing proteins do. This polyreactivity may have facilitated the emergence of a powerful regulatory system. As is exquisitely illustrated in immune network theory [11–13], Abs can also function as antigens and as such they may stimulate the production of second-class Abs—where ‘class’ refers to a set of Abs with a certain idiotype—if some threshold concentration is exceeded. Second-class Abs could in turn stimulate the production of third-class Abs and so on. In this chain of reaction Ig domain-containing proteins reversibly bind and thereby block each other. Thus, even if present, Ab-like proteins may operate at minimal levels or may lie dormant, leaving their ligands unbound. In cases when e.g. third-class Abs are stimulated and block second-class Abs, first class Abs could efficiently respond to their ligands [11–13]. It is worth noting that if we consider how widespread Ig domain-containing proteins are in nature, it is possible that a similar regulatory system predated the evolution of jawed vertebrates.

The Ab-like proteins that are described above would have features found in existing NAAb_B-1a_. Moreover, the expression of a diverse repertoire of Ab-like proteins may enable a broad range of interactions between Abs and self-components and provokes the emergence (or the amplification) of a system that regulates these interactions.

## LINKING ANTIBODY RESPONSE TO INNATE IMMUNITY

The emergence (or the optimization) of a system for the clearance of Abs-antigen complexes would bring us a step further toward the modern AIS. In enabling the removal of Abs that are bound to e.g. remnants of apoptotic or necrotic cells or conserved patterns of commensal microbiota, this system would link the components of the IIS to the low-affinity Abs-producing system. B-1 (but not B-2) cells are able to phagocytose large particles and bacteria, though not as extensively as macrophages [90–92]. Thus, a clearance system might have already been in place. Alternatively, Fc receptors, which interact with the constant region of Abs and appeared at the base of the bony fishes [93], might have played a significant role in this clearance system.

## LINKING THE PRODUCTION OF NAABS TO THE PRODUCTION OF ANTIGEN-SPECIFIC ABS

**Figure 1.**
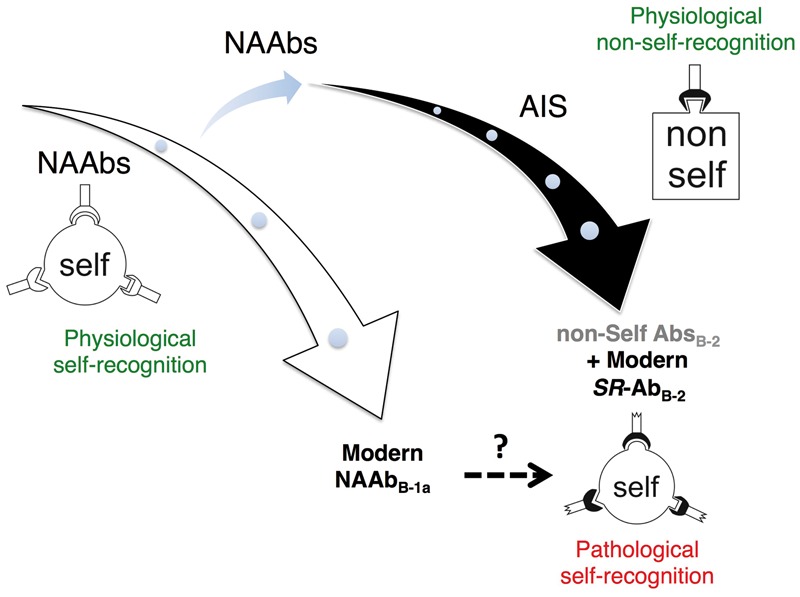
Hypothesis for the origin of the AIS in jawed vertebrates. Subsequent to the whole genome duplication that predated the radiation of jawed vertebrates (blue arrow), the AIS—a non-self-recognition system—gradually emerged from a regulated self-recognition system that is presently part of the IIS and produces natural autoantibodies via B-1a cells (NAAb_B-1a_). A population-genetic environment wherein the power of random genetic drift exceeds the power of selection might have favored the emergence of the AIS. NAAb_B-1a_ are physiologically produced; they contribute to tissue homeostasis and protect from pathological self-reactivity. *SR*-Ab_B-2_ are the AIS’s counterpart of NAAb_B-1a_. They can cause pathological self-reactivity and are normally counter-selected during the production of B-2 cell-derived non-self-targeting Abs (non-Self Abs_B-2_). It still remains unclear whether pathological self-reacting Abs result from misregulated B-1a cells, B-2 cells or subgroups thereof. Furthermore, the primary source of pathological self-reacting Abs may vary depending upon the types of AD

The interactions described thus far involve Ab-like proteins that (i) are partly self-reactive, (ii) are encoded by germ-line, unmutated or minimally mutated V(D)J gene sequences and (iii) have low affinity for their antigens. We postulate that the regulated production of non-self-reactive and high-affinity Abs emerged from this immune environment, subsequent to the whole genome duplication (WGD) that occurred before the radiation of jawed vertebrates [15, 94–97] (Fig. 1). The process of somatic hypermutation by the evolution of activation-induced cytidine deaminase from RNA-editing enzymes, affinity maturation and clonal selection would be paramount for achieving higher levels of Ab affinity. Post-WGD neofunctionalization of one or both copies of duplicated genes and/or events of subfunctionalization (i.e. partition of functions between copies of duplicated genes) could have facilitated the emergence of these AIS components, even in the absence of adaptive benefits [98–100]. This hypothetical scenario predicts that members of duplicate-gene pairs might display distinct patterns of expression in B-1a and B-2 cells. Although waiting for this prediction to be formally tested, we note that several mouse genes with duplicated copies [101] are expressed differentially between B-1 cells and B-2 cells [102].

**Figure 2.**
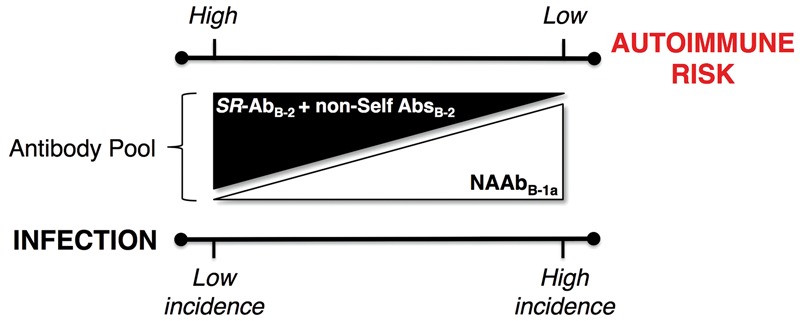
We propose (i) that *SR*-Ab_B-2_ alongside non-Self Abs_B-2_ were originally produced from NAAb_B-1a_-like receptors and (ii) that the present-day production of B2-cell-derived Abs is enhanced in environments where NAAb_B-1a_’ production—chiefly taking place during fetal/neonatal period—and/or activity are reduced. Acting as first line of defense, the production of NAAb_B-1a_ is enhanced in environments with a high incidence of infection. This excess of NAAb_B-1a_ protects from ADs whereas the limited production of *SR*-Ab_B-2_ reduces the risk for ADs. In environments with a low incidence of infection the relative excess of *SR*-Ab_B-2_ alongside the reduction of NAAb_B-1a_ enhance the risk of developing ADs

A central idea of our hypothesis is that *SR*-Ab_B-2_ and non-self-recognizing Abs were evolutionarily generated not only from but also ‘at the expense’ of NAAb_B-1a_. This implies that the production of high-affinity non-self-recognizing Abs was (and might still be) facilitated in an environment where the production/activity of NAAb_B-1a_ is reduced, i.e. an environment where the host suffers from a lowered ability to avoid autoimmunity and where the first line of defense against infections is weakened. This idea is consistent with observed hyperactivity of the AIS in diseases with autoimmune components [103] and with the observation that autoimmunity—a condition that can result from a low titer of NAAb_B-1a_—has enhancing effects on the fine-tuning of the adaptive immune responses [104]. Furthermore, this idea predicts that the enhanced production/activity of NAAb_B-1a_, e.g. in response to relatively heavy pathogen loads, protects from autoimmunity and inhibits or delays the production of *SR*-Ab_B-2_ and antigen-specific Abs (Fig. 2). These predictions align well with the hygiene hypothesis, which posits that the lowered exposure to infectious agents (particularly in early life) is the basis for increasing incidence of both autoimmune and allergic diseases [14, 105–107]. Noteworthy, the mechanisms underlying the hygiene hypothesis center on the antagonizing effects of T-helper cells (Th1/Th17 and Th2) [108] The theoretical framework that we propose support and further extend these mechanisms, given the role that B1-cells play in stimulating and regulating T-cell responses [109].

Which factors might have determined the suggested reduction in production/activity of early NAAb_B-1a_ in the ancestor of jawed vertebrates? One factor could be gene duplication, which may be coupled with a substantial reduction in expression level of each copy compared to the progenitor gene [110, 111]. Another factor could be a population-genetic environment where the power of random genetic drift exceeds the power of selection. In this environment mildly deleterious mutations, which can fix in small populations and might be selected secondarily [112], are expected to accumulate and duplicated genes to be preserved more easily by subfunctionalization [98, 99]. These accrued mutations could have weakened the existing IIS—including the system that produced natural autoantibody-like receptors —thereby imposing selection pressures for novel defense mechanisms with fitness benefits that outweigh the costs of inadequate immune responses. These fitness effects are compatible with empirical studies in sheep, chicken, and human where autoantibody production—whether physiological or reflecting antibody responsiveness is difficult to conclude firmly—has been found to scale positively with survival [113–118]. In sum, it is conceivable that a drift-dominated population-genetic environment in the ancestor of jawed vertebrates could have facilitated (i) the preservation of duplicated genes, (ii) the weakening of the mechanisms that control self-recognition and/or regulated the production/activity of NAAb-like receptors and (iii) the emergence of the somatic hyper mutation machinery.

## SELF- AND NON-SELF-RECOGNITION, THROUGH EVOLUTIONARY TIME

The arguments laid out until this point provide a reasonable and expandable framework which explains how the AIS could have gradually emerged in the ancestor of jawed vertebrates and may continue to operate today. Arguments that support or challenge aspects of our hypothesis may be established through an extended examination of existing accredited immunological models. Natural Abs can recognize self, altered self, and foreign antigens. This ability might be testimony to the proposed evolutionary link between self-reactivity and adaptive immunity. Self-antigens can be altered (e.g. due to oxidative stress [119]) and become immunogenic. Additionally, many natural Abs that react with altered self-antigen recognize epitopes expressed on pathogens. In this context, an examination of the altered-self antigen model [120, 121] might provide valuable insight on the early evolution of Abs and the regulation of self and non-self antigen recognition.

Insights offered by appropriate systems may be leveraged to further assess other aspects of our hypothesis. Pregnancy may be one such system. Given that pregnancy evolved much later than the AIS (probably <∼250 My ago [122, 123]), it may provide minimal new knowledge about the early evolution of AIS. However, because of the intimate relationship between pregnancy and immunity, pregnancy presents itself as a powerful system for scrutinizing the hypothesis that the present-day production of high-affinity non-self-recognizing Abs and the production/activity of NAAb_B-1a_ are interconnected. Because the genomes of the placenta and fetus are partially of paternal origin, immune tolerance—which bears on the responses to self- and non-self antigens [124]—is absolutely required for normal pregnancy to unfold. When these peculiar immunological circumstances are in place, pregnancy provides a natural laboratory for studying the relationships between IIS and AIS as well as these systems’ plasticity. Given that the AIS response is weakened during pregnancy whereas the IIS response is boosted [125–130], our hypothesis specifically predicts that the production/activity of NAAb_B-1a_ is enhanced during the course of a normal pregnancy, in line with previous studies [128, 131]. We expect that this relative excess of NAAb_B-1a_ (i) mitigates autoimmunity and (ii) inhibits/delays the production/activity of non-self-recognizing Abs and hence of potentially harmful *SR*-Ab_B-2_. Indeed, ADs may regress during normal pregnancy only to reappear in the post-partum phase [132]. For example, rheumatoid arthritis and multiple sclerosis often attenuate during normal pregnancy, only to re-aggravate after childbirth [133–136]. Pregnant women, on the other hand, seem to be relatively more susceptible to some infectious diseases, such as listeriosis and influenza [137–139]. We also expect that in women with an abnormally strong adaptive immune response against fetal and placental antigens (e.g. in egg-donation pregnancies [140])—note that in these conditions our hypothesis predicts an intensified production of *SR*-Ab_B-2_ alongside a lower-than-normal production/activity of NAAb_B-1a_—the likelihood of pregnancy-related pathologies with autoimmune components, such as recurrent pregnancy loss, preeclampsia and preterm delivery, should increase as is indeed observed [141–145]. Finally, because physiological high levels of progesterone may inhibit the antigen-presentation function of B cells, an aspect that should facilitate implantation and pregnancy [146], we expect that artificially enhancing the levels of progesterone in the maternal body could dually hinder the adaptive immune response [146] and help treat high-risk pregnancies with autoimmune components, in line with previous observations [147–150]. In sum, the maternal immune system does not simply shut down to promote the tolerance of the fetus. Rather, it adopts a peculiar state. In this new state, the relationships between the self- and non-self-recognition systems are largely consistent with the theoretical framework that we propose.

## A FRAMEWORK FOR TESTABLE PREDICTIONS

Our hypothesis provides other testable predictions, some of which (listed below) may provide a mechanistic explanation of a number of observations.

First, intravenous immunoglobulins (IVIg) are commercial soluble preparations obtained from human sera pooled from a broad number of healthy donors. B-1a cell-derived NAAbs constitute a considerable part of Ig in humans and are part of these preparations [151]. Provided that NAAb_B-1a_ truly down-regulate the production of high-affinity antigen-specific Abs (and thus *SR*-Ab_B-2_), IVIg should help counter inflammation and autoimmune disorders. In line with this idea, the therapeutic preparation of IVIg often has positive effects on individuals who suffer from autoimmunity [152]. With regard to semi alloimmunity (i.e. the condition of the fetus presenting only partially foreign (paternal) antigens) and pregnancy, we may further expect that experimental protocols based on IVIg application alleviate diseases with possible autoimmune components such as recurrent abortion, preterm delivery and preeclampsia [153].

Second, if B-1a cell-derived NAAbs have a homeostatic function that predates the emergence of the AIS, homeostatic perturbations might be detected in Ig-deficient jawed vertebrates such as severe combined immunodeficiency (SCID) mice. The altered expression of cytokines in the serum of SCID-mice compared with wild-type mice, for example, may be one of these perturbations [154]. In contrast, the loss of somatic hypermutation in an otherwise functional AIS background should not exhibit such homeostatic alterations. Additionally, specific alterations of the immune repertoire in animals such as the axolotl, a salamander that has a diverse antibody repertoire but fails to mount efficient adaptive immune responses [155], could shed light on alternative functions of NAAb_B-1a_.

Third and last, it has been found that dysbiosis may activate the AIS [69]. Based on our hypothesis, this finding implies that the production/activity of NAAb_B-1a_ may decrease in hosts with altered microbiota. Thus, dysbiosis might increase the risk for developing ADs. Enhancing the production/activity of NAAb_B-1a_ in hosts with altered microbiota should offset the hyperactive AIS and ward off or mitigate existing ADs. The administration of probiotics, which are proposed to elevate the production of NAAb_B-1a_ [156], might be beneficial in this regard. Likewise the exposure to sufficiently large pathogen loads should, in theory, also be advantageous. This latter prediction is in line with the rationale for and the proposed beneficial effects of the helminth therapy [157].

## AN EXPANDABLE HYPOTHESIS

The conceptual framework summarized here makes fair assumptions about how current data and theories can be interpreted and integrated coherently towards a better understanding of AIS evolution. We believe that our propositions may be useful to further expand current views on the origins and the evolution of the innate and AISs. Further efforts are still required, however, to account for the role of other immunological players such as TCRs and the MHC, which is involved in antigen presentation and regulation of B- and T-cell activity during immune responses, or Ig-isotypes (IgM, IgG, IgA etc.), whose different roles at specific life stages have not been extensively discussed here. Self-reactive Abs may be beneficial, merely decorative or pathogenic and self-reactivity needs to be considered through the prism of functional outcome. In addition, ADs are often a compilation of symptoms with potentially different ontologies, which makes it difficult to ascribe effects of a particular molecular agent on a specific disease. Finally, published observations largely stem from studies of mammals, with little knowledge yielded on other clades of vertebrates. All these knowledge gaps and limitations will be at least partly overcome in the future and new developments would serve to assess the validity of our hypothesis.

## CONCLUSIONS

We have presented a hypothesis for the origins and the evolution of the AIS in jawed vertebrates, where a self-recognition system alongside mechanisms that prevent pathogenic self-reactivity and do not elicit T-dependent responses predated and were instrumental for the emergence of a non-self-recognition system. Our hypothesis leverages and extends previous arguments and experimental observations concerning a number of evolutionary and medical aspects that have been published in the past few decades. In addition to making specific predictions, our hypothesis coherently integrates propositions about the evolutionary origin of the AIS with (i) current knowledge about NAAbs, (ii) concepts drawn from the ‘immune network’ and the ‘immunological homunculus’ theories, (iii) the 2 R hypothesis, (iv) mechanisms of gene duplicate preservation, (v) the ‘hygiene hypothesis’ and (vi) clinical observations and characteristics of pregnancy at the same time. Our simple hypothesis reconciles and bridges experimental and theoretical arguments that for long time have lived apart. It provides a framework for interpreting recent and less recent observations and offers potentially useful guidelines for future experiments.
